# Retrospective Evaluation of Central Venous Catheter Use for Parenteral Nutrition in Pediatric Intestinal Failure: Infections and Taurolidine Role

**DOI:** 10.3390/antibiotics15020193

**Published:** 2026-02-10

**Authors:** Júlia Vicentin de Souza, Angelica Sczepaniak da Silva, Lucas Gabriel Souza da Silva, Jéssica de Carvalho Inácio, Meire Ellen Pereira, Luíza Siqueira de Lima, Jaqueline de Sousa Fortes, Thaís Muniz Vasconcelos, Libera Maria Dalla Costa, Jocemara Gurmini, Cláudia Sirlene Oliveira

**Affiliations:** 1 Instituto de Pesquisa Pelé Pequeno Príncipe, Av. Silva Jardim, 1632, Água Verde, Curitiba 80250-060, PR, Brazil; 2 Faculdades Pequeno Príncipe, Av. Iguaçu, 333, Rebouças, Curitiba 80230-020, PR, Brazil; 3 Hospital Pequeno Príncipe, R. Des. Motta, 1070, Curitiba 80250-060, PR, Brazil

**Keywords:** nutritional support, short-bowel syndrome, microbial identification, antimicrobial lock therapy

## Abstract

Objective: This study aimed to describe the main microorganisms causing catheter-related bloodstream infections (CRBSIs) and to evaluate the effectiveness of taurolidine catheter lock therapy in children with intestinal failure (IF) receiving parenteral nutrition (PN). Study design: This retrospective study included 31 pediatric patients with IF admitted between 2017 and 2022 who received PN via central venous catheters (CVCs). Demographic, clinical, and laboratory data were collected, along with information on PN use, catheter characteristics, and infection episodes, including clinical signs, microbiological cultures, and antimicrobial therapy. Serum C-reactive protein and albumin levels, as well as the use of taurolidine lock therapy, were analyzed. Results: The median age was 54.4 days among patients who developed CRBSI and 154.1 days among those without CRBSI. The median duration of PN was 119 days in patients with CRBSI and 89 days in those without. Nineteen patients experienced CRBSI, accounting for 55 infection episodes confirmed by blood cultures obtained from CVCs. The most frequently isolated microorganisms were *Staphylococcus epidermidis*, *Enterococcus faecalis*, and *Klebsiella pneumoniae*. Taurolidine lock therapy was significantly associated with lower infection rates per 1000 catheter days, with most infected catheters and infection episodes occurring in the absence of taurolidine use. Conclusions: These findings contribute to the characterization of the microbiological profile of CRBSIs in pediatric patients with IF and support the use of advanced preventive strategies, such as taurolidine lock therapy, to reduce infection rates in children receiving long-term PN.

## 1. Introduction

According to the European Society for Clinical Nutrition and Metabolism (ESPEN), intestinal failure (IF) is defined as a reduction in intestinal function to levels insufficient for the adequate absorption of macronutrients, water, and electrolytes, thereby requiring intravenous supplementation to maintain health and growth. The ideal diagnosis involves the use of balance study techniques to assess intestinal demand and absorptive capacity, providing greater diagnostic specificity. However, due to limitations within healthcare systems, such as the scarcity of specialized centers, the need for intravenous supplementation is frequently used as a pragmatic clinical criterion for defining IF [1,2].

In pediatric patients, short bowel syndrome (SBS) is the most prevalent cause of IF, typically resulting from extensive intestinal resections or congenital malformations [3]. In neonates, SBS is particularly common and is most often associated with necrotizing enterocolitis, intestinal atresia, or volvulus [4,5]. From a functional perspective, SBS is characterized by impaired absorption of both macronutrients and micronutrients, leading to compromised intestinal function [6]. Consequently, SBS is often accompanied by dependence on parenteral nutrition (PN) and poses significant clinical challenges, adversely affecting quality of life, increasing healthcare costs, and contributing to higher morbidity and mortality rates [6].

IF is the rarest form of organ failure, with an estimated incidence ranging from 14.1 to 56 cases per million children worldwide. In Brazil, approximately 800 cases per million inhabitants are estimated to occur annually [7]. However, the scarcity of specialized centers and the presence of underreporting hinder accurate assessment of the true incidence in the country. Moreover, epidemiological studies focusing on patients dependent on PN in Brazil remain limited, underscoring the urgent need for research initiatives and systematic patient registries to better characterize the disease burden and its impact [7].

PN plays a central role in this context by providing essential nutrients when intestinal absorption is compromised [8,9,10]. PN consists of a carefully balanced formulation of macronutrients, including proteins, carbohydrates, and lipids, as well as micronutrients, electrolytes, and water, tailored to meet individual patient requirements. It is administered through a central venous catheter (CVC), which may be inserted peripherally or tunneled for long-term use. Such vascular access requires meticulous care to prevent complications, including catheter-related bloodstream infections (CRBSIs) and thrombosis, which can adversely affect prognosis and limit the long-term feasibility of PN therapy [3].

Among the potential complications, CRBSIs are frequent in patients receiving PN, particularly in pediatric patients with SBS, due to prolonged dependence on CVCs. The incidence of CRBSIs ranges from 1.3 to 10.2 episodes per 1000 catheter-days and is higher in children under one year of age. These infections may result from contamination during catheter handling, inadequate care of the insertion site, or hub contamination. In pediatric patients with SBS, CRBSIs are commonly associated with enteric pathogens, including *Escherichia coli* and *Klebsiella* spp. [5].

For the diagnosis of CRBSIs, internationally recognized guidelines from the Clinical and Laboratory Standards Institute (CLSI) and the Infectious Diseases Society of America (IDSA) recommend obtaining blood cultures whenever CRBSI is suspected, allowing appropriate investigation and confirmation [10,11,12]. Whenever feasible, catheter salvage is preferred to minimize the risk of vascular access loss; therefore, in the absence of severe complications, CRBSIs may be managed without catheter removal through the use of systemic antimicrobial therapy and antimicrobial lock techniques. However, catheter removal is indicated in cases of severe, persistent, or complicated infections [6,13]. CRBSIs can lead to serious consequences, including recurrent sepsis, loss of vascular access, and, in extreme cases, death [6].

Advanced preventive strategies have proven effective in reducing the incidence of CRBSIs [14,15]. Among these, catheter lock therapy with taurolidine, a taurine derivative, exhibits broad antimicrobial activity against gram-negative bacteria (e.g., *Klebsiella pneumoniae*), Gram-positive bacteria (e.g., vancomycin-resistant *enterococci* and *Staphylococcus epidermidis*), and fungal species (e.g., *Candida* spp.). Taurolidine is approved by the ESPEN and, to date, no cases of bacterial resistance have been reported in the scientific literature [14,16].

A multidisciplinary approach involving specialized teams is essential for the early prevention and management of these infections, thereby improving clinical outcomes [5,17]. In this context, given the clinical relevance of CRBSIs in pediatric patients dependent on PN and the scarcity of specific data, this retrospective study aims to evaluate CVC use for PN in children with IF, identify the main microorganisms responsible for CRBSIs, and assess the effectiveness of taurolidine catheter lock therapy.

## 2. Results

### 2.1. Clinical Characteristics of the IF Patients

**Figure 1:**
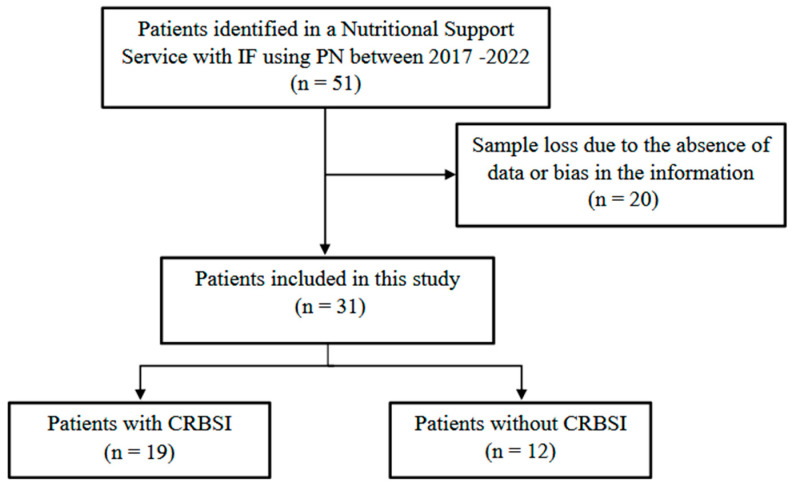
Flowchart of patient identification, screening, and inclusion in the study.

Of the 51 patients assessed, 31 met the inclusion criteria and were included in the study, while 20 were excluded due to missing data or information bias (Figure 1). The demographic and clinical characteristics of the 31 patients are summarized in Table 1. Of these, 19 developed CRBSI, while 12 did not; statistical analyses were performed accordingly. The Mann–Whitney U test revealed a significant difference in age between patients who developed CRBSI and those who did not (U = 63.50; *p* = 0.0399). Patients with CRBSI had a lower median age (54.4 days) compared with those without CRBSI (154.1 days).

Regarding sex distribution, among patients with CRBSI, 8 (42.1%) were female and 11 (57.9%) were male. In the group without CRBSI, 5 patients (41.7%) were female and 7 (58.3%) were male. Regarding underlying diagnoses, SBS was present in 14 patients with CRBSI (73.7%), while 5 (26.3%) had IF due to other etiologies. Among patients without CRBSI, 6 (50.0%) had SBS and 6 (50.0%) had IF due to other causes. SBS was the predominant cause of IF, most commonly secondary to necrotizing enterocolitis, intestinal atresia, volvulus, and other surgical conditions. Data on residual bowel length and ostomy status were inconsistently reported and were not analyzed.

Patients who developed CRBSI used a total of 96 CVCs, whereas patients without CRBSI used 36 CVCs. The Mann–Whitney U test demonstrated a significant difference in the number of CVCs used per patient between groups (U = 62.00; *p* = 0.0322). The CRBSI group had a higher median number of catheters per patient (median = 4) compared with patients without CRBSI (median = 3). Catheter characteristics differed between groups. In patients with CRBSI, double-lumen catheters were most frequently used (47.92%), whereas Broviac catheters predominated among patients without CRBSI (41.67%). In both groups, the jugular vein was the most common site of catheter insertion (59.38% vs. 61.11%). Patients with CRBSI had longer median durations of PN and catheter use compared with patients without CRBSI (119 vs. 89 days and 157 vs. 104 days, respectively).

Of the 31 patients included in the study, 19 experienced at least one episode of infection, accounting for a total of 55 CRBSI episodes (mean of 3 episodes per patient; range: 1–9; Supplementary Table S1). These episodes occurred across 50 catheters, reflecting the recurrent nature of CRBSIs in this population and supporting the use of episode- and time-based analyses. Among the 55 CRBSI episodes, 19 (34.55%) occurred during the use of taurolidine lock therapy, whereas 36 (65.45%) occurred without lock therapy. The 55 episodes were associated with 50 catheters due to the occurrence of multiple infections in some patients using the same catheter. In contrast, 12 patients experienced no infection episodes and used a total of 36 catheters, of which 20 (55.56%) were managed with taurolidine lock therapy and 16 (44.44%) without lock therapy.

#### 2.1.1. Signals and Symptoms of CRBSIs

Fever was the most frequent symptom in CRBSI episodes both with and without taurolidine lock therapy (Table 2). However, episodes occurring without taurolidine were characterized by a broader diversity of clinical manifestations. In this group, fever was followed by higher incidences of purulent discharge at the catheter insertion site, vomiting, hypoactivity, tachycardia, and diarrhea. In contrast, episodes associated with taurolidine use presented a narrower spectrum of symptoms; after fever, the most frequently reported signs were vomiting, diarrhea, hypoglycemia, and abdominal distension (Table 2). Owing to the retrospective nature of the study, detailed and standardized records of local catheter insertion site findings were not consistently documented in the medical charts. Consequently, it was not possible to further stratify these findings according to catheter type.

#### 2.1.2. Microorganisms: Culture, Incidence, and Resistance Patterns

Blood cultures were most frequently obtained concomitantly from peripheral veins and catheters (45.45%), followed by cultures collected exclusively from the catheter (38.18%). Additional combinations of microbiological sampling included peripheral blood and catheter tip cultures (7.27%); peripheral blood, catheter blood, and catheter tip cultures (3.64%); peripheral blood, catheter tip, and catheter secretion cultures (1.82%); catheter blood with catheter tip cultures (1.82%); and peripheral blood, catheter blood, and catheter secretion cultures (1.82%) (Supplementary Table S1).

Regarding the observed microorganism patterns, infection episodes were categorized according to the use of taurolidine lock therapy (Table 3). A greater diversity of microorganisms was observed in episodes occurring without taurolidine. *Staphylococcus epidermidis* was the most frequently isolated microorganism in episodes with taurolidine use, whereas *Enterococcus faecalis* predominated in episodes without taurolidine (Table 3). Episodes attributed to coagulase-negative *staphylococci* were classified as CRBSI only when accompanied by a compatible clinical presentation and evidence of an inflammatory response.

Antimicrobial resistance was identified in several bacterial species (Supplementary Table S1). Oxacillin resistance was observed among multiple staphylococcal species (*S. hominis*, *S. epidermidis*, *S. haemolyticus*, and *S. saprophyticus*), predominantly in *S. epidermidis*. In addition, *K. pneumoniae* isolates exhibited diverse resistance profiles, including probable extended-spectrum β-lactamase (ESBL) production and probable serine carbapenemase production (Ambler class A), with the detection of the bla-KPC gene confirmed by a reference laboratory. Probable serine carbapenemase production (Ambler class A) was also observed in *P. aeruginosa*. Furthermore, *S. aureus* isolates showed probable presence of the mecA gene, conferring resistance to oxacillin and most cephalosporins (except ceftaroline), consistent with a methicillin-resistant *S. aureus* (MRSA) phenotype.

#### 2.1.3. Taurolidine Blocked Therapy

A statistically significant difference in the use of taurolidine lock therapy was observed between patients with and without CRBSI (Chi-square test, *p* = 0.0034; Table 1). In the CRBSI group, most catheters were used without taurolidine lock therapy, with 69 of 96 catheters (71.87%) lacking taurolidine and only 27 (28.13%) receiving the therapy. In contrast, in the non-CRBSI group, the majority of catheters were treated with taurolidine lock therapy, with 20 of 36 catheters (55.56%) receiving taurolidine and 16 (44.44%) used without it. When infection episodes were analyzed, 36 of 55 episodes (65.45%) occurred in the absence of taurolidine lock therapy, whereas 19 episodes (34.55%) occurred during its use. These results underscore that this episode-based analysis is distinct from, yet complementary to, the catheter- and patient-based analyses.

**Figure 2:**
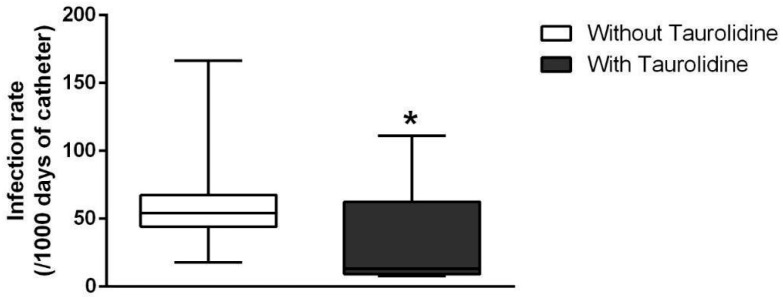
Infection rate per 1000 catheter days with and without taurolidine lock therapy. Data were analyzed using the Mann–Whitney U test and are presented as median (interquartile range). n = 11 with taurolidine and n = 14 without taurolidine. * Significantly different from the group without taurolidine (*p* < 0.05).

When infection rates per 1000 catheter days were analyzed, the Mann–Whitney U test revealed a statistically significant difference between groups (U = 39.50; *p* = 0.0428). The median infection rate without taurolidine lock therapy was 54.09 infections per 1000 catheter days, compared with 13.07 infections per 1000 catheter days in the taurolidine group (Figure 2). Overall, the infection rate was 25.07 infections per 1000 catheter days when taurolidine use was not considered.

#### 2.1.4. Analysis of Albumin and CRP

**Figure 3:**
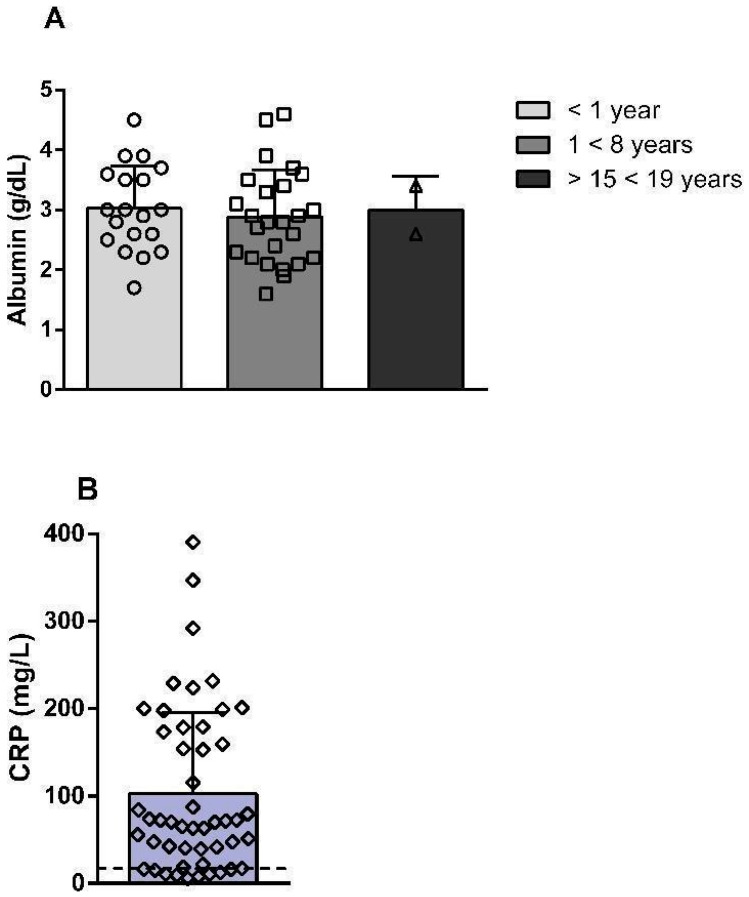
Distribution of albumin (**A**) and C-reactive protein (CRP) (**B**) values during CRBSI episodes in pediatric patients with intestinal failure. Reference ranges for serum albumin were 2.9–4.9 g/dL for patients aged 15 days to <1 year, 3.9–4.9 g/dL for those aged 1 to <8 years, and 4.2–5.3 g/dL for those aged 15 to <19 years. The reference value for CRP was <10 mg/L.

The mean serum albumin level across the 55 infection episodes was 3.04 ± 0.66 g/dL, while the mean CRP level was 93.38 ± 71.50 mg/L (Supplementary Table S1). Figure 3 illustrates the distribution of albumin (A) and CRP (B) values across CRBSI episodes. For albumin, age-specific reference values were considered, revealing reduced levels in patients aged 1 to <8 years and 15 to <19 years compared with their respective reference ranges. In contrast, albumin levels in patients younger than 1 year, although borderline in some cases, remained within the reference range. Regarding CRP, most values were above the reference range, consistent with an inflammatory response during CRBSI episodes.

#### 2.1.5. Antimicrobial Therapy

A total of 15 different antibiotics and one antifungal agent were prescribed during the study period. Among antibiotics, vancomycin was the most frequently prescribed (21 prescriptions), followed by meropenem (13 prescriptions) (Supplementary Table S1). Micafungin was the only antifungal agent prescribed, accounting for three prescriptions. In addition, prescription information was not available in the medical records for six cases.

#### 2.1.6. Clinical Outcome

Regarding the clinical outcomes of the 19 patients who experienced infection episodes, eight deaths (42.10%) were recorded, including two attributable to septic shock. Eight patients (42.10%) achieved intestinal rehabilitation, two (10.53%) were referred for home PN, and one patient (5.26%) remained hospitalized at the end of follow-up. Notably, three of the eight patients who died had been transferred to another hospital before death. The two deaths due to septic shock were associated with CRBSI caused by *K. pneumoniae*; one isolate was a probable ESBL producer, and the other a probable serine carbapenemase producer (Ambler class A) (Supplementary Table S1).

## 3. Discussion

This study evaluated the use of CVC in patients with IF receiving PN, identified the main microorganisms associated with CRBSIs, and assessed the efficacy of taurolidine lock therapy. The results indicate that SBS was the primary underlying cause of IF in this cohort, corroborating findings from previously published studies [15,18,19]. With respect to CVC use for PN, this study indicates that patients who developed CRBSIs required a greater catheter burden than those without infection, reflecting increased vascular access demands in this subgroup. These findings are consistent with the notion that recurrent catheter use may be both a marker and a contributor to infection risk in patients with IF. In comparison, Akaishi et al. [20], in a single-center retrospective study conducted over eight years (2011–2019) in pediatric patients with SBS, reported a lower catheter burden, with a mean of 1.5 CVCs per patient. Taken together, these observations suggest that the cohort analyzed in the present study experienced a higher intensity of catheter utilization over a shorter observation period, which may partially explain the elevated susceptibility to catheter-related complications. In line with the greater catheter burden observed among patients who developed CRBSI, catheter characteristics also differed between infected and non-infected groups. In the present cohort, catheters with higher functional complexity, such as double-lumen devices, were more commonly used in patients with CRBSI, whereas tunneled single-lumen catheters (e.g., Broviac and Hickman) predominated among patients without infection. Across both groups, the jugular vein was the preferred insertion site, reflecting standard practice in pediatric IF. These findings are consistent with previous reports. Chan et al. [18] observed a predominance of single-lumen catheters in children with IF and identified the jugular vein as the site most frequently requiring repair, accounting for approximately 53% of cases. Similarly, Reigadas et al. [21] reported the jugular vein as one of the most commonly used insertion sites in their cohort. Collectively, these observations suggest that catheter type and insertion site selection may influence catheter longevity and infection risk, particularly in patients requiring repeated or prolonged vascular access.

With regard to CRBSIs, the most prevalent microorganisms identified in this study were *S. epidermidis*, *K. pneumoniae*, and *E. faecalis*. These findings are consistent with previous reports identifying *S. epidermidis* as a leading Gram-positive pathogen in CRBSIs, largely due to its capacity to form biofilms that promote adherence to catheter surfaces and persistence within the intraluminal environment [22]. In a single-center study from Japan evaluating blood culture profiles in patients with CRBSI, *S. epidermidis* was likewise reported as the most frequently isolated microorganism [20]. *K. pneumoniae*, a commensal organism of the gastrointestinal tract, is also widely recognized as a clinically relevant gram-negative pathogen in CRBSIs, particularly because of its ability to form biofilms on catheter surfaces [11,23]. Previous studies have demonstrated a higher prevalence of gram-negative bacteria in peripheral vascular access-related infections compared with central access-related infections (33% vs. 18.8%, respectively) [24]. Moreover, virulence factors such as the polysaccharide capsule and lipopolysaccharides, along with the acquisition of antimicrobial resistance genes, contribute to the therapeutic challenges associated with *K. pneumoniae*-related CRBSIs [23]. Notably, in patients with SBS, CRBSIs are frequently associated with enteric microorganisms, including *Klebsiella* spp., reinforcing the relevance of these findings in this specific population [5]. The incidence of *E. faecalis*-associated CRBSI, a Gram-positive bacterium that is part of the normal intestinal microbiota, has increased in recent years. Similar to *S. epidermidis* and *K. pneumoniae*, *E. faecalis* has the capacity to form biofilms on catheter surfaces, facilitating persistence and recurrent infection. Moreover, the expression of antimicrobial resistance genes by this species poses an additional therapeutic challenge [25]. Reigadas et al. [21] characterized enterococcal CRBSI as an emerging clinical entity, reporting *Enterococcus* spp. as the fourth most frequent cause of CRBSI in their institution and demonstrating that at least 6% of enterococcal bacteremia episodes were catheter-related. Notably, approximately 85% of CRBSI episodes caused by *Enterococcus* spp. in that study were attributed to *E. faecalis*.

Among the three main pathogens identified in this study, antimicrobial resistance patterns were observed in *S. epidermidis* and *K. pneumoniae*, reinforcing the clinical relevance of these organisms in CRBSI. Oxacillin resistance in *S. epidermidis* is of particular concern, as oxacillin is traditionally considered a first-line agent for infections caused by coagulase-negative *staphylococci*. Resistance to this antimicrobial has been associated with worse clinical outcomes, including increased morbidity and mortality [26]. Consistent with this, Pereira and Cunha [26] reported a high prevalence of the mecA gene among oxacillin-resistant coagulase-negative *staphylococci* isolated from neonatal blood cultures, with *S. epidermidis* representing the majority of resistant strains. Broader evidence supports the prominence of *S. epidermidis* as a major cause of CRBSIs. A systematic review by Siciliano et al. [27] demonstrated that coagulase-negative *staphylococci*, particularly *S. epidermidis*, account for approximately 30–40% of bloodstream infections, most of which are associated with CVCs. The authors further highlighted mecA-mediated resistance as the predominant mechanism underlying oxacillin resistance in these infections. In this context, linezolid has emerged as an alternative therapeutic option, with documented success in cases of bacteremia caused by methicillin-resistant *S. epidermidis*, despite limited large-scale clinical data [27,28].

Resistance among *K. pneumoniae* isolates represents an even greater therapeutic challenge, particularly in pediatric patients with IF. Previous studies have shown that *Klebsiella* spp. are major contributors to healthcare-associated and CRBSIs, with ESBL production being frequently reported and strongly associated with adverse outcomes, including increased mortality [29]. In addition, carbapenem-resistant *K. pneumoniae* has emerged as a critical concern in CRBSI, as highlighted by Abdulall et al. [30], who reported high rates of carbapenem resistance among gram-negative isolates from CRBSI in intensive care settings. Taken together, these findings underscore the dual challenge posed by biofilm-forming capacity and antimicrobial resistance in CRBSI pathogens, particularly *S. epidermidis* and *K. pneumoniae*, and highlight the importance of preventive strategies and judicious antimicrobial stewardship in vulnerable pediatric populations.

CRBSI remains a major complication among patients requiring long-term central venous catheterization, with reported incidence rates varying widely according to the population studied and the diagnostic criteria applied [5,15,22,31]. In pediatric cohorts, reported CRBSI rates generally range from 1.3 to 10.2 episodes per 1000 catheter days, with higher incidences observed in children younger than one year of age [5]. However, the lack of uniformity in CRBSI definitions across studies has contributed to substantial heterogeneity in reported rates, complicating direct comparisons and leading to discrepancies in epidemiological estimates [32]. Within this context, the present findings underscore the clinical burden of CRBSIs in pediatric patients with IF and highlight the potential protective role of taurolidine lock therapy. The lower frequency of taurolidine use among catheters associated with infection, together with the significant reduction observed in infection rates when adjusted for catheter exposure time, supports an association between taurolidine use and reduced CRBSI occurrence. This association is particularly relevant in the setting of infections caused by biofilm-forming microorganisms, as biofilm matrices confer protection against host immune responses and antimicrobial agents, thereby facilitating persistent and recurrent infections [32]. The antimicrobial effect of taurolidine is mediated by its degradation products, which interact with microbial cell wall components and inhibit biofilm formation, offering a mechanistic rationale for its preventive use in catheter care [1,14,33]. Consistent with these observations, Ling et al. [15] reported a substantial reduction in pathogen-related infection episodes following the introduction of taurolidine lock therapy, with an approximate 44% decrease in overall infection rates. Collectively, these data reinforce the role of taurolidine lock therapy as an effective strategy for reducing CRBSI risk in vulnerable pediatric populations requiring long-term central venous access.

To strengthen the diagnosis of true CRBSI, this study integrated microbiological findings with clinical manifestations and inflammatory biomarkers, including CRP and albumin. Fever emerged as the most consistent clinical feature, reinforcing its value as a key indicator of CRBSI. This observation is in line with previous reports in both adult and pediatric IF populations. Dibb et al. [34], in a long-term prospective study conducted in a national IF unit, identified pyrexia as the most frequent presenting symptom of CRBSI. Similarly, Robinson et al. [35] demonstrated that fever was the most reliable clinical sign at CRBSI onset in children with IF. Together, these findings support the use of fever, in conjunction with laboratory biomarkers, as an important component in confirming clinically relevant CRBSI and distinguishing true infection from contamination.

With respect to laboratory markers, CRP is a well-established indicator of systemic inflammation and plays an important role in innate immune responses, including processes such as opsonization and phagocytosis. Elevated CRP levels are commonly observed during the acute phase of inflammatory and infectious conditions [11,36]. Serum albumin, although traditionally used as a nutritional marker, is also strongly influenced by inflammation and acute illness, with concentrations typically declining during inflammatory states [37]. In the present study, the combined assessment of elevated CRP and reduced albumin (interpreted according to age-specific reference ranges) provided supportive evidence for active infection when considered alongside clinical manifestations and microbiological findings. This approach aligns with previous reports in patients with IF. Sakurai et al. [37] demonstrated a significant inverse relationship between serum albumin levels and CRBSI rates in patients receiving PN, while Robinson et al. [35] identified CRP as the only laboratory parameter consistently associated with CRBSI onset among several inflammatory and hematological markers.

The clinical outcomes observed in patients with CRBSI episodes in the present study highlight the substantial severity of these infections in individuals with IF receiving parenteral nutrition. Although mortality attributable directly to septic shock was limited, the overall burden of adverse outcomes appears considerable when contrasted with long-term single-center experiences. For example, Hojsak et al. [38], in a 21-year retrospective study predominantly involving pediatric patients with SBS, reported only two deaths due to septic shock. When viewed in relation to the much shorter observation period of the present study, this comparison underscores the potential impact of CRBSIs on morbidity and mortality in this vulnerable population and reinforces the clinical importance of effective preventive and management strategies.

This study has several inherent limitations. Its retrospective, single-center design restricts the generalizability of the findings and limits control over data completeness and standardization. Detailed information on catheter care practices, indications and timing for catheter replacement, duration and selection of antimicrobial therapy, catheter removal versus salvage strategies, and specific components of PN was not consistently documented in the medical records and therefore could not be systematically evaluated. Additionally, although serum albumin and CRP were included as laboratory markers because of their availability, albumin has limited sensitivity to acute nutritional changes and was interpreted in the context of systemic inflammation and overall clinical status rather than as a marker of acute malnutrition. Finally, the retrospective nature of the study precluded determination of the predominant source of CRBSI, such as exogenous contamination during catheter manipulation versus endogenous bacterial translocation from the gastrointestinal tract. Despite these limitations, the present findings reinforce the clinical relevance of CRBSI prevention in pediatric patients with IF and support the implementation of rigorous catheter care protocols. Moreover, the observed associations highlight the potential benefit of advanced preventive strategies, including antimicrobial lock therapies, as part of a comprehensive approach to optimizing the management and outcomes of CRBSIs in this vulnerable population.

## 4. Materials and Methods

### 4.1. Clinical Setting, Study Design and Patients

This retrospective study evaluated medical records from a cohort of pediatric patients (n = 51), aged 0–19 years, diagnosed with IF who were admitted to and received PN at a large Brazilian pediatric hospital in Curitiba, Paraná, between 2017 and 2022. This hospital serves as a national referral center, providing care to pediatric patients from Curitiba as well as from other regions of the country, which explains the relatively high number of cases (n = 51) observed despite the low prevalence of IF. The study population comprised exclusively hospitalized pediatric patients who were receiving PN at the time of CRBSI occurrence. Patients undergoing home parenteral nutrition were explicitly excluded from the analysis. CVC handling and maintenance were conducted exclusively by trained nursing personnel in accordance with standardized institutional protocols.

Data were extracted from the hospital’s electronic medical record system and included patient characteristics (sex, age, and underlying diagnosis), PN-related variables (catheter type and insertion site, duration of catheter use, and duration of PN), CRBSI-related information (clinical signs and symptoms, identified microorganisms from blood cultures, and antimicrobial therapy), and laboratory parameters (C-reactive protein and serum albumin levels). Episodes of CRBSI were systematically described, and the effect of taurolidine catheter lock therapy was also evaluated. Owing to the retrospective nature of the study, detailed information on specific PN components and additives was not consistently available in the medical records and, therefore, was not included in the analysis.

All patient data were anonymized prior to analysis and entered into a secure computerized database. The study protocol was approved by the hospital’s ethics committee (approval no. 6267487). Informed consent was waived in accordance with ethical guidelines due to the retrospective nature of the study.

#### Inclusion Criteria

The study included pediatric patients aged 0–19 years with IF who were hospitalized and received PN via a CVC during the period from 2017 to 2022.

### 4.2. Diagnosis of CRBSI Episodes

Microbiological evaluation was primarily based on blood cultures, with catheter tip and catheter lumen secretion cultures analyzed when available in the medical records. Specimen collection was performed following strict aseptic techniques and standardized institutional procedures by the hospital nursing staff, according to medical orders, and samples were subsequently processed by the hospital’s clinical laboratory. Episodes of CRBSI were defined based on positive blood culture results in conjunction with compatible clinical signs and symptoms.

To define episodes of CRBSI, internally standardized criteria were applied based on the time interval between positive blood cultures and catheter replacement. Positive blood cultures yielding the same microorganism from the same catheter within an interval of 7 to 15 days were classified as persistent infection and considered a single CRBSI episode. Conversely, positive blood cultures isolating the same microorganism from different catheters with an interval greater than 15 days were classified as distinct infection episodes. These time intervals were established in accordance with the Diagnostic Criteria for Healthcare-Associated Infections issued by the National Health Surveillance Agency [39]. Additionally, blood cultures isolating two or more microorganisms from the same collection route were classified as a single polymicrobial CRBSI episode.

Given the high prevalence of coagulase-negative staphylococci as skin commensals, particular care was taken to distinguish true CRBSIs from potential contamination, especially in episodes involving *Staphylococcus epidermidis*. CRBSI was not defined based solely on a single positive blood culture. Instead, microbiological findings were interpreted in conjunction with clinical signs and symptoms, inflammatory markers (elevated C-reactive protein and/or hypoalbuminemia), initiation of antimicrobial therapy, and clinical course. In cases in which peripheral blood cultures were unavailable, positive catheter-drawn cultures were considered indicative of CRBSI only when supported by concordant clinical and laboratory evidence. This integrated approach was adopted to minimize the misclassification of contamination as true CRBSI.

Therefore, in our institution, all PN solutions are compounded in a centralized pharmacy unit under strict aseptic conditions and laminar airflow, adhering to international safety standards. Consequently, intrinsic contamination of the PN solution is considered an exceptionally rare event. For this reason, routine culturing of PN bags is not performed, and the diagnosis of bacteremia is based on differential time to positivity or simultaneous cultures from the central line and peripheral vein, focusing on catheter colonization rather than infusate contamination. 

### 4.3. Incidence—Signal and Symptoms of Infection and Microorganisms

Incidence was calculated as the number of occurrences of each clinical sign, symptom, or microorganism per 1000 CRBSI episodes. In this analysis, CRBSI episodes constituted the unit of analysis, and the denominator was used to standardize the frequency of events between groups with differing numbers of infection episodes (with and without taurolidine use). This incidence measure is descriptive in nature and reflects the distribution of findings across CRBSI episodes rather than patient-level or time-dependent risk estimates.

### 4.4. Infection Rate

To enhance the robustness and interpretability of the analysis, infection rates were calculated as the number of CRBSI episodes per 1000 catheter-days. For this purpose, infection episodes were stratified according to the use or nonuse of taurolidine catheter lock therapy. In addition, an overall infection rate per 1000 catheter-days was calculated for the study population with CRBSI, irrespective of taurolidine use.

### 4.5. Taurolidine Lock Treatment Protocol

Taurolidine lock therapy was not applied systematically to all patients and was restricted to those with long-term CVCs. In clinical situations requiring catheter replacement, such as suspected or confirmed CRBSI, patients frequently underwent temporary insertion of short-term CVCs before placement of a new long-term device. Owing to the retrospective design of the study, detailed information regarding the timing of catheter exchanges and the specific clinical indications for initiation of taurolidine lock therapy was not consistently documented in the medical records and therefore could not be analyzed in detail.

TauroLock™ (Bavaria, Germany) is one of the catheter-locking solutions used at the institution. It consists of (cyclo)-taurolidine combined with 4% citrate and exhibits bactericidal, fungicidal, and anticoagulant properties [5,33]. Following medical prescription and authorization for dispensing TauroLock™, the solution is administered into the central venous catheter (CVC) by the nursing staff in accordance with the institution’s standardized internal protocol, which is described step by step below:

1. Perform hand hygiene according to the institutional standard operating procedure;
2. Disinfect the CVC connections using an alcohol-based antiseptic, applying friction for at least 10 s prior to access;
3. Flush the catheter with 10 mL of 0.9% saline solution;
4. Slowly instill the ready-to-use taurolidine solution to completely fill the catheter lumen and, at the end of administration, simultaneously close the extension clamp or, when applicable, the catheter body using a positive-pressure technique;
5. Allow the taurolidine solution to remain within the catheter lumen until the next treatment or for a minimum dwell time of 2 h;
6. Before the subsequent catheter use, aspirate and discard the taurolidine solution from the catheter lumen and flush the catheter with 0.9% saline solution.

In patients with IF receiving PN, the catheter lumen used for taurolidine lock administration alternates daily in order to maintain uninterrupted PN infusion. The lock lumen is changed once every 24 h. In patients with single-lumen catheters, the taurolidine lock is administered during PN-free intervals, ensuring a minimum dwell time of 2 h before the subsequent PN infusion.

### 4.6. Albumin and C-Reactive-Protein (CRP)

Serum albumin and CRP concentrations were measured in blood samples collected during routine clinical care and retrieved retrospectively from the patients’ medical records. Laboratory analyses were performed in the hospital’s accredited clinical laboratory using standardized analytical procedures and internal quality control measures. Results were interpreted according to the institution’s validated reference ranges. Age-specific reference intervals for serum albumin were 2.9–4.9 g/dL for patients aged 15 days to <1 year, 3.9–4.9 g/dL for those aged 1 to <8 years, and 4.2–5.3 g/dL for adolescents aged 15 to <19 years. The reference value for CRP was consistent across age groups and defined as <10 mg/L.

### 4.7. Antimicrobial Therapy

Data on antimicrobial use were extracted from the medical records of patients who experienced CRBSI episodes. Antimicrobial therapy initiated after positive blood culture results was recorded, and treatment data were updated following receipt of antimicrobial susceptibility testing (antibiogram) and/or antifungal susceptibility results. When available, susceptibility profiles indicating antimicrobial or antifungal resistance were also documented.

Owing to the retrospective design of the study, detailed data on antibiotic treatment duration, catheter management strategies (removal versus salvage), and treatment outcomes related to catheter preservation were not consistently documented in the medical records and, therefore, could not be systematically analyzed.

### 4.8. Statistical Analysis

Data were organized using Microsoft Excel spreadsheets. Descriptive statistics were selected according to variable type. Continuous variables were summarized as medians and interquartile ranges, whereas categorical variables were presented as absolute frequencies and percentages. For inferential analyses, Fisher’s exact test, the Chi-square test, and the Mann–Whitney U test were applied as appropriate, based on data distribution and homogeneity. All statistical analyses were performed using GraphPad Prism software (version 5.0). A *p*-value < 0.05 was considered statistically significant.

Analyses were conducted at different levels according to the research question. Patient-level analyses were used to describe demographic and clinical characteristics. Catheter-level analyses were performed when comparing infected and non-infected catheters. Episode-level analyses were used to describe microbiological findings, clinical signs and symptoms, and antimicrobial resistance patterns, with each CRBSI episode considered an independent clinical event. Infection rates were calculated per 1000 catheter-days to account for time at risk. Owing to the retrospective design, limited sample size, and exploratory nature of the study, clustering of observations within individual patients was not formally modeled. This limitation is acknowledged and should be considered when interpreting the results, particularly given the occurrence of multiple infection episodes in the same patient.

## 5. Conclusions

In this retrospective study, *S. epidermidis*, *K. pneumoniae*, and *E. faecalis* emerged as the most frequent pathogens associated with CRBSIs, contributing to a better understanding of the local microbiological profile. The use of taurolidine lock therapy was associated with a significant reduction in infection rates per 1000 catheter days, underscoring its potential role in CRBSI prevention. Although mortality was limited, fatal outcomes due to septic shock caused by *K. pneumoniae* highlight the clinical severity of these infections in patients with IF receiving PN. Overall, these findings contribute to characterizing patterns of CVC use and infection in this population and support the implementation of advanced preventive strategies, including antimicrobial lock therapies, to optimize catheter management and patient outcomes.

## Figures and Tables

**Table 1:** Patients, parenteral nutrition (PN), and central venous catheter (CVC) characteristics.

Characteristics	CRBSI (n = 19)	No CRBSI (n = 12)	*p*-Value
Age [days; median (interquartile interval)]	54.4 (29.0–189.5)	154.1 (107.0–577.9) *	0.0399 ^a^
Sex			
Female [n (%)]	8 (42.1)	5 (41.7)	1.0000 ^b^
Male [n (%)]	11 (57.9)	7 (58.3)	
Underlying disease			
SBS [n (%)]	14 (73.7)	6 (50.0)	0.2553 ^b^
IF by other causes [n (%)]	5 (26.3)	6 (50.0)	
CVC (n)	96	36	-
CVC/patients [median (interquartile interval)]	4 (3–6)	3 (2–4) *	0.0322 ^a^
Taurolidine look therapy			
CVC with taurolidine [n (%)]	27 (28.1)	20 (55.6)	0.0034 ^c^
CVC without taurolidine [n (%)]	69 (71.9)	16 (44.4)	
Days with catheter [median (interquartile interval)]	157 (74.0–227)	104 (89.0–228.3)	0.5286 ^a^
Days on PN [median (interquartile interval)]	119 (68.0–174.0)	89 (68.2–158.3)	0.4059 ^a^
Catheter Type [n (%)]			
Double-Lumen	46 (47.9)	6 (16.7)	-
Broviac	17 (17.7)	15 (41.7)	-
Hickman	8 (8.3)	10 (27.8)	-
Picc	14 (14.6)	4 (11.1)	-
Power-Picc	8 (8.3)	0	-
Simple CVC	1 (1.04)	1 (2.8)	-
External CVC	1 (1.04)	0	-
Port-a-Cath	1 (1.04)	0	-
Catheter localization—vein [n (%)]			
Jugular	57 (59.48)	22 (61.1)	-
Femoral	15 (15.6)	5 (13.9)	-
Subclavian	10 (10.4)	7 (19.4)	-
Cephalic	4 (4.1)	1 (2.8)	-
Axillary	2 (2.1)	0	-
Basilica	2 (2.1)	0	-
Brachial	1 (1.0)	0	-
Brachiocephalic	1 (1.0)	0	-
Saphenous	1 (1.0)	0	-
Not informed	3 (3.1)	1 (2.8)	-

* statistic different from CRBSI group; ^a^ Mann–Whitney U test; ^b^ Fisher test; ^c^ Chi-square test.

**Table 2:** Signals and Symptoms of CRBSIs expressed as incidence per 1000 episodes of infection.

Signals and Symptoms	Incidence (/1000 Episodes of Infection)	
	Without Taurolidine (n = 36)	With Taurolidine (n = 19)
Fever	888.88	526.31
Subfebrile	55.55	-
Hypothermia	27.77	52.63
Edema	-	52.63
Hyperemia around the catheter	27.77	52.63
Purulent discharge at insertion site	222.22	-
Ataxia	55.55	-
Bradycardia	55.55	-
Tachycardia	83.33	52.63
Apnea	27.77	-
Hypoglycemia	-	105.25
Desnaturation	27.77	-
Dehydration	0	52.63
Hipoactivity	111.11	52.63
Vomit	138.88	263.15
Diarrhea	83.33	210.52
Bloody stools	-	52.63
Abdominal flaccidity	55.55	-
Abdominal distension	-	105.26
Cyanosis	27.77	-
Sweating	27.77	-
Cardiorespiratory arrest	55.55	-
Hemodynamic instability	27.77	-
Sepsis	55.55	52.63
Septic shock	55.55	52.63

**Table 3:** Incidence of microorganisms per 1000 infection episodes in CRBSI episodes with and without taurolidine.

Microorganism Infection	Episode of Infection (n)		Incidence (/1000 Episodes of Infection)	
	Without Taurolidine	With Taurolidine	Without Taurolidine	With Taurolidine
*Staphylococcus epidermidis*	4	6	111.11	315.78
*Klebsiella pneumoniae*	5	4	138.88	219.52
*Enterococcus faecalis*	7	0	194.44	-
*Staphylococcus hominis*	4	1	111.11	52.63
*Escherichia coli*	2	2	55.55	105.26
*Staphylococcus aureus*	3	0	83.33	-
*Paenibacillus* spp	0	2	-	105.26
*Candida albicans*	2	0	55.55	-
*Candida haemuloni*	1	0	27.77	-
*Candida parapsilosis*	1	0	27.77	-
*Enterococcus faecium*	1	0	27.77	-
*Staphylococcus saprophyticus*	1	0	27.77	-
*Staphylococcus haemolyticus*	1	0	27.77	-
*Pseudomonas aeruginosa*	1	0	27.77	-
*Streptococcus oralis*	0	1	-	52.63
Infections with more than one microorganism	3	3	83.33	157.89
Total of episodes	36	19	-	-

## Data Availability

The original contributions presented in the study are included in the article/Supplementary Material. Further inquiries can be directed to the corresponding author.

## Supplementary Materials

The following supporting information can be downloaded at: https://www.mdpi.com/article/10.3390/antibiotics15020193/s1, Table S1: Raw data per patient.

## Abbreviations

The following abbreviations are used in this manuscript:

CRBSI	Catheter-Related Bloodstream Infection
CVC	Central Venous Catheter
CRP	C-Reactive Protein
ESBL	Extended-Spectrum Beta-Lactamase
IF	Intestinal Failure
MRSA	Methicillin-Resistant *Staphylococcus aureus*
NICU	Neonatal Intensive Care Unit
PN	Parenteral Nutrition
SBS	Short Bowel Syndrome
VRE	Vancomycin-Resistant Enterococci
