# Supplementary material for: Retrospective Evaluation of Central Venous Catheter Use for Parenteral Nutrition in Pediatric Intestinal Failure: Infections and Taurolidine Role
Source: Antibiotics (Basel). 2026 Feb 10;15(2):193. doi: 10.3390/antibiotics15020193 (PMC12937225; doi:10.3390/antibiotics15020193)
Supplement: Supplementary file 1 [file antibiotics-15-00193-s001.zip › antibiotics-4057540-supplementary.pdf]

Supplementary Material

Retrospective Evaluation of Central Venous Catheter use for Parenteral Nutrition in Pediatric Intestinal Failure: Infections and Taurolidine Role

Júlia Vicentin de Souza[a,b], Angelica Sczepaniak da Silva[b], Lucas Gabriel Souza da Silva [b], Jéssica de Carvalho Inácio[c], Meire Ellen Pereira[a,b], Luíza Siqueira de Lima[a,b], Jaqueline de Sousa Fortes[a], Thaís Muniz Vasconcelos[a,b], Libera Maria Dalla Costa[a, b] , Jocemara Gurmini[c], and Cláudia Sirlene Oliveira[a,b*]

[a] Instituto de Pesquisa Pelé Pequeno Príncipe, Curitiba, PR, Brazil.

[b] Faculdades Pequeno Príncipe, Curitiba, PR, Brazil.

[c] Hospital Pequeno Príncipe, Curitiba, PR, Brazil

*Corresponding authors:

Oliveira, C. S. (claudia.sirlene@professor.fpp.edu.br)

Instituto de Pesquisa Pelé Pequeno Príncipe

Supplementary Table S1 – Raw data per patient.

**Patient**	**CVC (Type)**	**Days of CVC**	**Taurolidine**	**Etiologic Agent (Microorganism)**	**Culture**	**CRP (mg/L)**	**Albumin (g/dL)**	**Antimicrobial**	**Possible Resistance**	**Outcome**
A1	BROVIAC	94	No	*Candida albicans (1x)*	Catheter and Limb (Blood Culture)	47.5	2.3	Micafungin	-	Death - septic shock - cardiorespiratory arrest
	DOUBLE LUMEN	10	No	*Enterococcus faecalis (1x)*	Catheter (Blood Culture)	47.5	2.3	Ampicillin	-	
	DOUBLE LUMEN	6	No	*Klebsiella pneumoniae (1x)*	Catheter (Blood Culture)	42.3	2.6	Not informed	Positive phenotypic test for probable production of Serine-Carbapenemase (Ambler Class A).	

A3	DOUBLE LUMEN	20	No	*Klebsiella pneumoniae (1x)*	Catheter and Limb (Blood Culture)	39.3	3.9	Vancomycin and meropenem	Strain-producing extended-spectrum beta-lactamase (ESBL) should be considered, according to the CLSI (Clinical and Laboratory Standards Institute), resistant to penicillins, cephalosporins, and aztreonam.	Death - Septic shock - Gasping and cardiorespiratory arrest.
A4	DOUBLE LUMEN	7	No	*Escherichia coli (1x)*	Catheter (Blood Culture)	346.5	2.3	Piperacillin + tazobactam	-	Rehabilitated
	DOUBLE LUMEN	29	Yes	*Staphylococcus hominis (1x); Staphylococcus epidermidis (1x)*	Catheter and Limb (Blood Culture);	10.4; Not informed; Not informed	2.9; Not Informed; Not Informed	Vancomycin; Vancomycin; Piperacilin + tazobactam	*S. hominis and S. epidermidis* - Isolates with resistance to oxacillin should be	

				and Escherichia coli (1x)	Catheter and Limb (Blood Culture); Catheter (Blood Culture) and Catheter Tip (Culture)			and meropenem	considered, according to CLSI 2018 (Clinical and Laboratory Standards Institute), resistant to all beta-lactam antibiotics (penicillins, cephalosporins, and carbapenems).	
	DOUBLE LUMEN	32	No	*Escherichia coli (1x)*	Catheter (Blood Culture)	18.6	3.5	Meropenem and then de-escalation to cefepime	-	
A9	PICC	4	No	*Candida Haemuloni (1x)*	Catheter and Limb (Blood Culture)	390.6	Not informed	Not informed.	-	Rehabilitated

DOUBLE LUMEN	21	No	*Enterococcus faecalis (1x)*	Catheter and Limb (Blood Culture) and Catheter Tip (Culture)	159.4	Not informed	Ciprofloxacin and Ampicillin.	-
HICKMAN	9	Yes	*Klebsiella pneumoniae* (1x)	Catheter (Blood Culture)	228.9	2.2	Vancomycin and meropenem	-
HICKMAN	13	No	*Enterococcus faecalis (1x)*	Catheter and Limb (Blood Culture)	40.1	3.6	Ampicillin and gentamicin	-

	DOUBLE LUMEN	25	No	*Staphylococcus hominis (1x)*	Catheter and Limb (Blood Culture)	16.2	Not informed	Vancomycin and meropenem	Isolates with resistance to oxacillin should be considered resistant to all beta-lactam antibiotics (penicillins, cephalosporins, and carbapenems)	
A11	DOUBLE LUMEN	29	No	*Enterococcus faecalis* (1x)	Catheter and Limb (Blood Culture)	200.1	2.0	Ampicillin, Gentamicin and Cefepime	-	Transferred/Death. Non-exclusive palliative care - vascular failure
	PICC	47	No	*Klebsiella pneumoniae (1x)*	Catheter and Limb (Blood Culture)	70.0	2.9	Polymyxin B	Resistant to meropenem, gentamicin, ciprofloxacin, ampicillin, cefipime. Genes related to	

								carbapenemase production were not detectable, they were researched: bla-KPC and bla-NDM.
DOUBLE LUMEN	38	No	*Staphylococcus epidermidis, Enterococcus faecium e Klebsiella oxytoca (1x)*	Catheter and Limb (Blood Culture)	199,3	2,8	Daptomycin + Meropenem + Polymyxin B	Staphylococci with resistance to oxacillin should be considered resistant to all beta-lactam antibiotics (penicillins, cephalosporins and carbapenems)
POWERPICC	64	Yes	*Staphylococcus epidermidis (1x)*	Catheter (Blood Culture)	179.1	2.7	Vancomycin	Staphylococci with resistance to oxacillin should be considered resistant to all beta-

								lactam antibiotics (penicillins, cephalosporins and carbapenems)
DOUBLE LUMEN	51	No	*Klebsiella pneumoniae (1x)*	Catheter and Limb (Blood Culture)	154.1	Not informed	Meropenem and Amikacin	Positive phenotypic test for production of broad-spectrum beta-lactamase (ESBL), the enzyme is capable of hydrolyzing penicillins, cephalosporins up to fourth generation, and aztreonam.
DOUBLE LUMEN	117	No	*Staphylococcus epidermidis (2x)*	Catheter and Limb	55.5; 178.5	3.0; 2.6	Piperacilin + tazabactam and vancomycin	Staphylococci with resistance to oxacillin should be considered

				(Blood Culture)				resistant to all beta-lactam antibiotics (penicillins, cephalosporins, and carbapenems)	
	POWERPICC	77	No	*Enterococcus faecalis (1x)*	Catheter and Limb (Blood Culture)	87.2	Not informed	Ampicillin and Gentamicin	-	
A15	PICC	46	No	*Staphylococcus epidermidis (1x)*	Catheter (Blood Culture)	16.4	Not informed	Cefepime	Isolates with resistance to oxacillin should be considered, according to CLSI 2018 (Clinical and Laboratory Standards Institute), resistant to all beta-lactam antibiotics (penicillins,	Death. Cardiorespiratory arrest.

								cephalosporins and carbapenems).
BROVIAC	319	Yes	*Staphylococcus hominis and Klebsiella pneumoniae (1x); Enterococcus faecalis and Staphylococcus haemolyticus (1x); Klebsiella Pneumoniae (2x)*	Catheter and limb (Blood Culture); Catheter (Blood Culture); Catheter and limb (Blood Culture); Catheter (Blood Culture).	152.8; 70.4; 41.6; 17.0	2.1; 2.2; 2.8; 1.9	*S. hominis and K. pneumoniae* – aztreonam, ceftazidime + avibactam + daptomycin. *E. faecalis e S. haemolyticus* – Vancomycin. *K. pneumoniae* - Cefepime, linezolid and ampicillin.	*S. hominis* - Isolates with resistance to oxacillin should be considered, according to CLSI 2018 (Clinical and Laboratory Standards Institute), resistant to all beta-lactam antibiotics (penicillins, cephalosporins and carbapenems). *K. pneumoniae* - Positive test for carbapenemase

K. pneumoniae - Ceftazidime + Avibactam.	production (detectable bla-KPC gene), test performed at LACEN/PR using Real-Time PCR (Taqman).
	K. pneumoniae - Positive phenotypic test for probable production of Serine-Carbapenemase, the enzyme hydrolyzes penicillins, cephalosporins, carbapenems and aztreonam, in addition to presenting decreased susceptibility to beta-

								lactams associated with beta-lactamase inhibitors (amoxacillin, clavulanic acid, piperacillin, tazobactam and ampicillin sulbactam).	
A19	DOUBLE LUMEN	19	No	*Pseudomonas aeruginosa and Serratia marcescens (1x)*	Catheter and Limb (Blood Culture)	64.9	3.4	Vancomycin	-	Transferred to another hospital and death - no information on the reason for death.
A20	BROVIAC	259	Yes	*Staphylococcus epidermidis (2x)*	Catheter and Limb (Blood Culture) and Catheter	Not informed; 72.5	1.6; 2.4	Vancomycin.	Isolates with resistance to oxacillin should be considered, according to CLSI 2018 (Clinical and Laboratory Standards Institute), resistant to all beta-	Rehabilitated

					(Blood Culture)				lactam antibiotics (penicillins, cephalosporins, and carbapenems).	
A21	POWERPICC	16	Yes	*Klebsiella pneumoniae (1x)*	Catheter and limb (Blood Culture) and catheter tip	231.4	2.5	Meropenem.	Strain producing extended-spectrum beta-lactamase (ESBL) and should be considered, according to the CLSI (Clinical and Laboratory Standards Institute), resistant to penicillins, cephalosporins and aztreonam.	Hospital discharge and after, death. No information on the reason for death.
	DOUBLE LUMEN	43	No	*Klebsiella pneumoniae (1x)*	Catheter (Blood Culture)	5.9	3.5	Not informed.	Positive test for probable	

									carbapenemase production.	
A22	EXTERNAL CATHETER	22	Yes	*Staphylococcus hominis and Staphylococcus epidermidis (1x)*	Catheter (Blood culture)	5.9	3.5	Piperacilin tazabactam and vancomycin	*S. epidermidis* - Isolates with resistance to oxacillin should be considered resistant to all beta-lactam antibiotics (penicillins, cephalosporins, and carbapenems).	Rehabilitated
A24	BROVIAC	16	No	*Klebsiella pneumoniae (1x)*	Catheter and Limb (Blood Culture)	291.9	1.7	Vancomycin and Gentamicin	Positive phenotypic test for probable production of Serine-Carbapenemase (Ambler Class A), the enzyme hydrolyzes penicillins, cephalosporins,	Death - cardiorespiratory arrest.

									carbapenems and aztreonam, in addition to presenting decreased susceptibility to beta-lactams associated with beta-lactamase inhibitors (amoxicillin, clavulanic acid, piperacillin, tazobactam and ampicillin subactam).	
A25	HICKMANN	125	Yes	*Paenibacillus spp (1x)*	Catheter (Blood Culture)	51.2	2.6	Not informed.	-	Domiciliar PN
A28	DOUBLE LUMEN	3	No	*Candida parapsilosis (1x)*	Limb (Blood Culture), catheter tip,	223.7	3.0	Micafungin	-	Rehabilitated

	DOUBLE LUMEN	9	No	*Staphylococcus hominis (1x)*	and catheter secretion Catheter (Blood Culture)	63.4	3.5	Not informed	-	
A29	DOUBLE LUMEN	18	No	*Staphylococcus haemolyticus (1x)*	Limb (Blood Culture) and catheter tip	201.0	2.8	Piperacilin tazabactam and vancomicyn	Isolates with resistance to oxacillin should be considered, according to CLSI 2018 (Clinical and Laboratory Standards Institute), resistant to all beta-lactam antibiotics (penicillins, cephalosporins and carbapenems).	Rehabilitated
	POWERPICC	43	Yes	*Staphylococcus epidermidis (1x)*	Catheter and limb	15.3	2.9	Vancomicyn	Isolates with resistance to oxacillin should be	

					(Blood Culture)				considered, according to CLSI 2018 (Clinical and Laboratory Standards Institute), resistant to all beta-lactam antibiotics (penicillins, cephalosporins, and carbapenems).	
	PICC	24	Yes	*Escherichia coli (1x)*	Catheter (Blood Culture)	84.1	2.2	Meropenem	-	
A36	DOUBLE LUMEN	13	No	*Staphylococcus aureus (1x), Pseudomonas aeruginosa (1x)*	Catheter and Limb (Blood Culture); Limb (Blood Culture)	197.9; 173.6	3.0; 2.6	*P. aeruginosa* – Vancomicyn. *S. aureus* – Vancomicyn and meropenem.	*S. aureus* - Development of Staphylococcus aureus MRSA. Probable presence of the mecA gene conferring resistance to oxacillin	Rehabilitated

					and catheter tip				and cephalosporins (except ceftaroline). *P. aeruginosa* - *Positive phenotypic test for probable production of Serine-Carbapenemase (Ambler Class A)	
A40	DOUBLE LUMEN	19	No	*Staphylococcus epidermidis (1x)*	Catheter (Blood Culture)	10.4	4.5	Piperacilin tazabactam	Isolates with resistance to oxacillin should be considered, according to CLSI 2018 (Clinical and Laboratory Standards Institute), resistant to all beta-lactam antibiotics (penicillins, cephalosporins, and carbapenems).	Rehabilitated

	HICKMANN	108	Yes	*Streptococcus oralis (1x)*	Catheter (Blood Culture)	71.6	3.7	Not informed	-	
A43	DOUBLE LUMEN	33	Yes	*Staphylococcus epidermidis (1x)*	Catheter and limb (Blood Culture)	73.6	3.0	Cefepime and vancomycin.	-	Domiciliar PN
	POWERPICC	120	Yes	*Paenibacillus spp (1x)*	Catheter (Blood Culture)	7.7	3.6	Cefepime and vancomycin.	-	
A44	DOUBLE LUMEN	34	No	*Staphylococcus aureus (1x)*	Catheter (Blood Culture)	Not informed	4.5	Piperacilin tazabactam	-	Death. Exclusive palliative care was chosen. Episodes of bradycardia and progression to death.
	DOUBLE LUMEN	26	No	*Staphylococcus hominis (1x)*	Catheter (Blood Culture)	Not informed	4.6	Piperacilin tazabactam	Staphylococci with resistance to oxacillin should be considered resistant to all beta-lactam antibiotics	

								(penicillins, cephalosporins, and carbapenems).
DOUBLE LUMEN	21	No	*Enterococcus faecalis (1x); Staphylococcus hominis (1x*)	Catheter (Blood Culture); Catheter (Blood Culture)	12.4; 9.1	3.9; 3.7	Ceftriaxone	*S. hominis* – Staphylococci with resistance to oxacillin should be considered resistant to all beta-lactam antibiotics (penicillins, cephalosporins, and carbapenems).
HICKMANN	10	No	*Staphylococcus aureus (1x)*	Catheter and limb (Blood Culture) and	Not informed	Not informed	Vancomycin and meropenem.	-

					Catheter secretion					
	DOUBLE LUMEN	18	No	*Enterococcus faecalis (1x)*	Catheter and limb (Blood Culture)	72.2	Not informed	Ampicilin and amikacin.	-	
	DOUBLE LUMEN	15	No	*Streptococcus parasanguinis, Pseudomonas aeruginosa and Staphylococcus aureus (1x)*	Catheter (Blood Culture)	63.5	3.3	Meropenem concentrate.	*P. aeruginosa* - Microorganism resistant to carbapenems. Negative phenotypic tests for carbapenemase production.	
A48	DOUBLE LUMEN	10	No	*Candida Albicans (1x)*	Catheter and Limb (Blood Culture)	21.9	3.1	Micafungin	-	The patient remains hospitalized.

DOUBLE LUMEN	19	No	*Staphylococcus saprophyticus (1x)*	Catheter and Limb (Blood Culture)	115.3	3.4	Meropenem and vancomycin	Staphylococci with resistance to oxacillin should be considered resistant to all beta-lactam antibiotics (penicillins, cephalosporins, and carbapenems).
SIMPLE CATHETER	18	No	*Enterococcus faecium (1x)*	Catheter and Limb (Blood Culture)	79.2	2.1	Linezolid and gentamicin.	-
